# Microneedle-based transdermal delivery systems for metabolic bone diseases: advances, challenges, and future perspectives

**DOI:** 10.3389/fbioe.2026.1793776

**Published:** 2026-02-25

**Authors:** Xingwen Xie, Xianli Zheng, Dingpeng Li, Qiang Zhou, Qiang Liu, Min Liu, Hui Wang, Naijia Liu, Yanping Zhu, Yongli Zhao, Yaxiong Gao

**Affiliations:** 1 Affiliated Hospital of Gansu University of Chinese Medicine, Lanzhou, China; 2 Gansu University of Chinese Medicine, Lanzhou, China; 3 The Second People’s Hospital of Gansu Province, Lanzhou, China

**Keywords:** bone-targeted nanocarriers, intelligent closed-loop delivery, metabolic bone diseases, microneedles, transdermal drug delivery

## Abstract

Metabolic bone diseases (MBDs), such as osteoporosis and rickets, present significant clinical challenges due to the chronic nature of treatment and the limitations of conventional systemic therapies. Oral medications often suffer from low bioavailability and gastrointestinal intolerance, while injectable biologics are hampered by poor patient adherence. Microneedle (MN) systems have emerged as a transformative transdermal delivery platform capable of overcoming these barriers. This review provides a comprehensive overview of MN technology, detailing its classification, material properties, and advantages in bypassing the stratum corneum for painless administration. We analyze how MNs have evolved from physical conduits into intelligent therapeutic platforms that integrate bone-targeting ligands, stimuli-responsive release mechanisms, and immunomodulatory functions to precisely regulate the bone microenvironment. Furthermore, we summarize recent preclinical advances in MN applications for MBDs, highlighting their ability to improve pharmacokinetic profiles and therapeutic efficacy. Finally, the review critically examines current hurdles regarding manufacturing, safety, and clinical translation, and offers perspectives on next-generation systems that combine diagnostic sensing with adaptive therapy to realize personalized bone health management.

## 1 Introduction

Metabolic bone diseases (MBDs) are a primary focus in medical research and clinical care. This category encompasses disorders such as osteoporosis, rickets and osteomalacia, osteogenesis imperfecta, Paget’s disease of bone, hypophosphatasia, and sclerosing bone dysplasias (Natesan and Kim, 2022). The etiologies of MBDs are highly heterogeneous. They range from nutritional deficiencies and systemic metabolic disturbances to pathogenic variants in bone-related genes. A central therapeutic challenge arises from the chronic nature of these conditions, as they disrupt the delicate equilibrium of bone remodeling governed by osteoblasts, osteoclasts, and osteocytes (Boyce et al., 2012). Currently, conventional pharmacotherapies face significant limitations. For instance, oral bisphosphonates suffer from poor bioavailability (typically <1%) due to limited gastrointestinal absorption (Fuggle et al., 2022; Saeting et al., 2022). They also cause esophageal irritation, leading to suboptimal patient adherence; nearly 50% of patients discontinue treatment within 1 year (Fuggle et al., 2022; Saeting et al., 2022). Similarly, injectable agents like parathyroid hormone (PTH) analogs require frequent subcutaneous administration. This often leads to injection-site reactions and further reduces adherence. Intravenous infusions are another option, but they necessitate clinical supervision, which increases both costs and infection risks (Rendina-Ruedy and Rosen, 2022). Consequently, there is an urgent need for innovative therapeutic strategies. Ideally, these new approaches should enhance efficacy while prioritizing patient-centered factors, such as ease of use and minimized side effects.

Microneedles (MNs) have emerged as a highly promising platform in tissue engineering and regenerative medicine (Dul et al., 2025; Zhang et al., 2023). In the context of metabolic disorders, MN-based transdermal drug delivery offers a minimally invasive alternative. This approach bypasses gastrointestinal and hepatic barriers while enabling controlled drug release (Razzaghi et al., 2024a; Zhang et al., 2024). MNs consist of arrays of micrometer-scale projections (100–1000 µm in height). They transiently penetrate the stratum corneum to create micropores, facilitating the direct delivery of therapeutics into the dermal microcirculation (Martínez-Navarrete et al., 2024). Importantly, this process does not activate cutaneous nociceptors, thereby ensuring a painless experience. Clinical exploration of MN patches for insulin and vaccine delivery has already demonstrated feasibility and improved adherence, offering a compelling precedent for MBD applications (Martínez-Navarrete et al., 2024). Furthermore, MNs overcome the molecular size limitations of conventional transdermal patches. For example, MNs integrated with nanoparticles can significantly enhance the permeation of macromolecules (Starlin Chellathurai et al., 2024). These systems are also highly versatile. They are available in various forms, including solid, coated, hollow, and hydrogel-forming varieties. This versatility allows for customized pharmacokinetics, ranging from rapid onset for acute fracture pain to sustained release for osteoporosis maintenance (Xue et al., 2024). Recent advances highlight their specific potential in metabolic disease therapy. Dissolving MNs made from biocompatible polymers, such as hyaluronic acid, have achieved >90% bioavailability in preclinical models of osteoporosis (Ma et al., 2023). Moreover, polymer-based MNs delivering bisphosphonates or PTH analogs show strong promise. They can mimic intermittent dosing profiles to promote osteoblast activity while suppressing osteoclast function (Ripolin et al., 2024; Zhang P. et al., 2025). Emerging designs even incorporate stimuli-responsive materials for biomarker-triggered drug release, aligning with the principles of precision medicine (Tian et al., 2025). Together, these interdisciplinary advances position MN technology as a transformative platform for bone health.

This review synthesizes the unmet needs and clinical constraints in current MBD management. We detail how microneedle-based transdermal delivery platforms offer a transformative solution to these challenges. Specifically, we discuss how MN systems have evolved from simple physical conduits to intelligent therapeutics. These advanced systems now combine targeted delivery, microenvironment modulation, and disease-modifying functions. We summarize the latest technological advances across various MBDs and examine how they improve delivery efficiency, enhance lesion specificity, and optimize therapeutic windows. Ultimately, these insights highlight the value of innovative MN systems in advancing MBD care toward precise, minimally invasive, and individualized treatment paradigms.

## 2 Classification of metabolic bone diseases by pathological metabolic pathways and associated drug delivery challenges

Although MBDs clinically manifest as impaired skeletal integrity, reduced mechanical strength, or abnormal mineral density, their root causes are not limited to local bone cell dysfunction. Instead, they reflect systemic metabolic imbalances that ultimately manifest in bone tissue. Consequently, viewing MBDs as a single disease entity is outdated. A more precise approach classifies them into distinct “pathological metabolic pathways” based on underlying molecular mechanisms. This framework supports accurate diagnosis and the development of highly targeted therapies.

### 2.1 A pathway-based classification framework for MBDs

Historically, MBDs were categorized by clinical phenotype or isolated etiologies. However, as the skeleton is increasingly recognized as an endocrine and metabolic organ, classification by core pathological pathways has become essential for understanding disease mechanisms and guiding precision interventions (Karner and Long, 2018; Karsenty and Khosla, 2022). Table 1 summarizes major MBD subtypes according to their dominant pathogenic pathways.

**TABLE 1:** Classification framework for the pathogenesis of metabolic bone diseases.

Category	Core mechanism	Representative conditions	Clinical features	First-line drugs/strategies	Monitoring essentials	References
Nutritional deficiency	Lack of calcium, phosphate, vitamin D	Rickets, osteomalacia, age-related osteoporosis	Often due to poor intake or malabsorption	Vitamin D (cholecalciferol/ergocalciferol), active vitamin D (calcitriol/alfacalcidol), calcium; phosphate if needed	Serum calcium, phosphate, 25-OH-vitamin D; urinary calcium if high doses; renal function	Baroncelli et al. (2024), Biasucci et al. (2024), Minisola et al. (2020)
Energy-metabolism impairment	Inadequate energy supply for bone remodeling (mitochondrial dysfunction)	Mitochondrial disease–related bone disease, chronic under-nutrition	Reduced osteoblast function, low bone formation	Optimize nutrition; treat osteoporosis per risk: bisphosphonates, denosumab, teriparatide/abaloparatide, romosozumab	Baseline and periodic calcium, renal function; dental risk assessment; DEXA every 1–2 years; consider CTX/P1NP to gauge response	LeBoff et al. (2022), Liu et al. (2024a), Suh and Lee (2024), Yan et al. (2023)
Mineral metabolism disorder	Ca–P imbalance, renal phosphate loss/retention	CKD-MBD (chronic kidney disease–mineral and bone disorder), hypophosphatemic rickets	Often with renal dysfunction, hyperphosphatemia	Phosphate binders (e.g., calcium salts, sevelamer), active vitamin D or analogs, calcimimetics (cinacalcet/etelcalcetide); XLH: burosumab (anti-FGF23)	Calcium, phosphate, PTH, alkaline phosphatase; for CKD-MBD follow KDIGO targets; for burosumab check fasting phosphate and dose-adjust; imaging for ectopic calcification if persistent hyperphosphatemia	Michigami et al. (2024), Thompson et al. (2025)
Hormonal dysregulation	Abnormal PTH, estrogen, thyroid hormones	Hyper/hypoparathyroidism, thyrotoxic bone disease, postmenopausal osteoporosis	High resorption or low formation	Postmenopausal osteoporosis: bisphosphonates first-line; denosumab; teriparatide/abaloparatide; romosozumab. Primary hyperparathyroidism: surgery or calcimimetic. Thyroid disease: treat the thyroid disorder; add bone agents per risk	Calcium, 25-OH-vitamin D; TSH/FT4 when thyroid disease present; PTH in parathyroid disorders; DEXA 1–2 years; fracture risk reassessment every 12–24 months	Lamy et al. (2025), Shoback et al. (2020)
Enzyme/transporter defects	Inborn errors affect mineral handling	XLH, osteopetrosis	Early onset; long-term therapy needed	XLH: burosumab. Malignant infantile osteopetrosis: hematopoietic stem-cell transplantation (HSCT); interim interferon-γ-1b in selected cases	XLH: fasting phosphate, ALP, growth and deformity tracking; dental evaluation. Osteopetrosis: hematologic profile, vision/hearing, imaging; HSCT protocol labs	Haffner et al. (2025), Nguyen et al. (2022), Liang et al. (2025)
Secondary to chronic disease	Primary systemic disease drives bone loss	Diabetic bone disease, cancer therapy–induced bone loss	Heterogeneous mechanisms; combination care needed	Optimize underlying disease; osteoporosis agents per risk. Cancer therapy–related bone loss (aromatase inhibitors/ADT): zoledronic acid or denosumab + calcium/vitamin D	Serum calcium, renal function; ONJ risk assessment and dental exam before potent antiresorptives; DEXA at baseline and 12–24 months	Ma and Zhang (2025), Vilaca and Eastell (2024), Gulyás et al. (2020)
Inflammatory/immune-mediated	Cytokines promote osteoclasts and inhibit formation	RA-, SLE-associated bone disease	Often with arthritis and bone erosion	Optimize disease control with biologics/targeted agents (anti-TNF, anti-IL-6R, JAK inhibitors, etc.). Add antiresorptives or anabolic therapy per fracture risk	Infection screening (TB, hepatitis B/C) before biologics; CBC, LFTs during therapy; DEXA 1–2 years; calcium/vitamin D status	Xu et al. (2023)

The first category comprises nutritional deficiency–related MBDs. These arise from inadequate intake, malabsorption, or abnormal metabolism of key nutrients, which directly impairs bone matrix mineralization or osteoblast function (Van Gastel and Carmeliet, 2021). Classic examples include osteomalacia in adults and rickets in children due to vitamin D deficiency. Vitamin D regulates intestinal calcium and phosphate absorption and influences the balance between osteoblast differentiation and RANKL/OPG signaling. Severe deficiency leads to defective mineralization of the osteoid, causing bone pain, fractures, and skeletal deformities (Sobh et al., 2022).

The second category involves disorders of mineral ion homeostasis, typically caused by dysregulated renal or intestinal handling of calcium, phosphate, or magnesium (Sprague et al., 2021). For instance, XLH results from PHEX gene mutations that impair renal phosphate reabsorption, leading to hypophosphatemia and defective bone mineralization (Méaux et al., 2022). Similarly, in CKD, reduced phosphate excretion causes hyperphosphatemia. This suppresses 1α-hydroxylase activity, lowers active vitamin D (1,25-(OH)2D) synthesis, and triggers secondary hyperparathyroidism, driving high-turnover bone disease (Tu et al., 2021). These conditions highlight the critical role of the gut–kidney–bone axis in mineral balance.

Furthermore, multiple endocrine hormones regulate bone remodeling equilibrium through direct or indirect pathways (Du Y. et al., 2021). Estrogen deficiency, common in postmenopausal women, leads to increased RANKL expression, decreased OPG, and enhanced osteoclast activation, constituting the primary mechanism of postmenopausal osteoporosis (Hooshiar et al., 2022). Conversely, long-term glucocorticoid exposure inhibits osteoblast proliferation, induces apoptosis, and promotes sclerostin expression. This suppresses the Wnt/β-catenin pathway and causes low-turnover bone loss (Marini et al., 2023). Other conditions, such as hyperthyroidism and abnormalities in the growth hormone/IGF-1 axis, also significantly impact bone turnover rates and quality (Mazziotti et al., 2022).

Simultaneously, bones serve not only as structural support but also as participants in energy metabolism. Insulin resistance, hyperglycemia, and dyslipidemia can impair bone health through multiple mechanisms (Armutcu and McCloskey, 2024). In diabetic bone disease, AGEs accumulate and disrupt collagen cross-linking. Meanwhile, hyperglycemia suppresses osteoblast function and alters the bone marrow microenvironment. Imbalances in adipokines (e.g., adiponectin, leptin) further disrupt remodeling equilibrium, increasing bone fragility even when bone density appears normal (Mangion et al., 2023). Immune-mediated mechanisms also contribute significantly to bone pathology. In chronic inflammatory states, pro-inflammatory cytokines directly target bone cells (Mi et al., 2024). In autoimmune conditions like RA and inflammatory bowel disease, persistently elevated TNF-α, IL-1, and IL-6 stimulate RANKL-driven osteoclastogenesis, causing periarticular bone erosion (Sonmez Kaplan et al., 2023). These cytokines also suppress osteoblast activity, leading to systemic bone loss. Such diseases underscore the importance of the immune–bone axis, or osteoimmunology, in MBD pathogenesis.

Finally, some MBDs result from monogenic mutations that directly disrupt bone matrix formation, mineralization, or cellular function, independent of nutritional or environmental factors. Examples include osteogenesis imperfecta (defective type I collagen synthesis), hypophosphatasia (ALPL mutations causing accumulation of mineralization inhibitors), and XLH (Claeys et al., 2021; Lim et al., 2017). Though rare, these disorders provide crucial insights into bone metabolism and serve as models for targeted therapies such as enzyme replacement or monoclonal antibodies.

**FIGURE 1:**
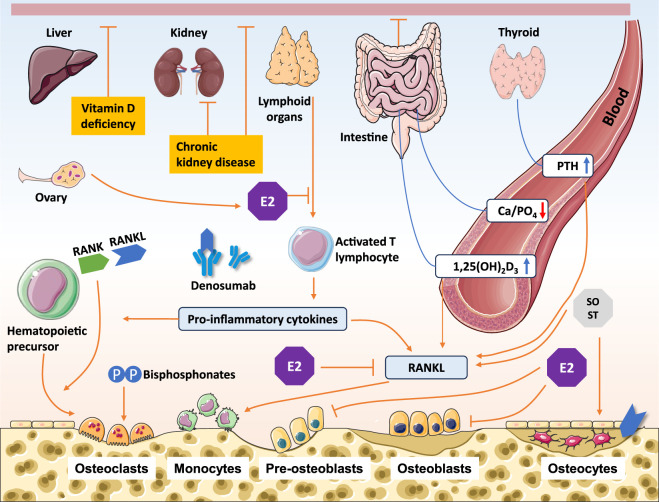
Pathogenic mechanisms and potential therapeutic targets in metabolic bone diseases. RANK, receptor activator of nuclear factor κB; RANKL, receptor activator of nuclear factor κB ligand; PTH, parathyroid hormone; SOST, sclerostin; 1,25(OH)_2_D_3_, 1,25-dihydroxyvitamin D_3_ (Calcitriol).

In summary, classifying MBDs by pathological metabolic pathways clarifies their heterogeneity. This approach provides a scientific foundation for shifting from symptomatic treatment to mechanism-based precision interventions (Figure 1).

### 2.2 Drug delivery challenges in MBDs therapy

Current mainstream drugs for MBDs, including small-molecule inhibitors and biologics, primarily rely on systemic administration. This presents significant pharmacokinetic and therapeutic challenges.

First, most drugs exhibit poor bone targeting (Lagunas-Rangel et al., 2024). After entering systemic circulation, they distribute widely, with only a tiny fraction penetrating the dense bone matrix to reach active remodeling sites like resorption lacunae or bone-forming surfaces. This inefficiency causes substantial off-target effects in non-skeletal tissues, increasing systemic toxicity risk (Verma et al., 2021). Second, the first-pass effect and low bioavailability pose major hurdles. For orally administered drugs, especially peptides or active vitamin D derivatives, gastrointestinal absorption is followed by rapid hepatic or intestinal metabolism and degradation. This reduces systemic concentrations below therapeutic levels, greatly lowering bioavailability (Lewiecki et al., 2008). Third, gastrointestinal intolerance limits several key medications. For instance, bisphosphonates are poorly absorbed and can irritate the gut, leading to esophagitis, gastric ulcers, and other effects that impair patient comfort and long-term adherence (Oliveira et al., 2024). Finally, patient adherence is a critical issue. As chronic diseases, MBDs require lifelong treatment, but prolonged high-frequency dosing, whether daily/weekly oral or subcutaneous injections, often reduces compliance, diminishing efficacy in reducing fracture risk.

**FIGURE 2:**
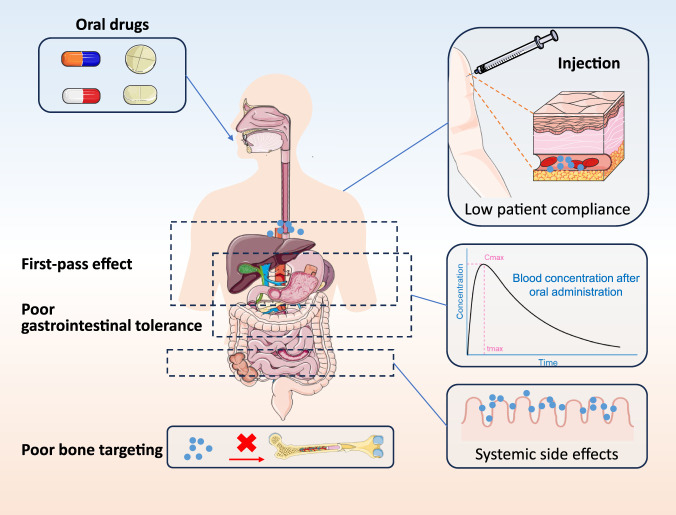
Various challenges affecting drug delivery and efficacy.

These delivery and pharmacokinetic barriers urgently demand novel smart drug delivery systems capable of overcoming biological obstacles and achieving targeted accumulation in the bone microenvironment (Figure 2).

## 3 Microneedle systems: a low-invasiveness, high-adherence transdermal strategy

MN systems represent a transformative platform for transdermal drug delivery. These systems utilize arrays of tiny needles to penetrate the outermost stratum corneum in a minimally invasive manner. With lengths ranging from several tens to several hundred micrometers, MNs are typically shorter than the depth of cutaneous nerve endings. Consequently, they create transient channels to the subdermal microcirculation without causing pain. This approach offers significant clinical advantages: it bypasses the gastrointestinal degradation and hepatic first-pass metabolism associated with oral dosing, and it can replace routine subcutaneous or intramuscular injections. Together, these features significantly improve patient adherence to treatment regimens.

### 3.1 Architecture and functional classes of MN systems

Based on their structural design and loading/release mechanisms, MN systems fall into five principal engineering types: solid microneedles (S-MNs), hollow microneedles (H-MNs), coated microneedles (C-MNs), dissolving microneedles (D-MNs), and hydrogel-forming microneedles (HF-MNs) (Liu L. et al., 2025).

**FIGURE 3:**
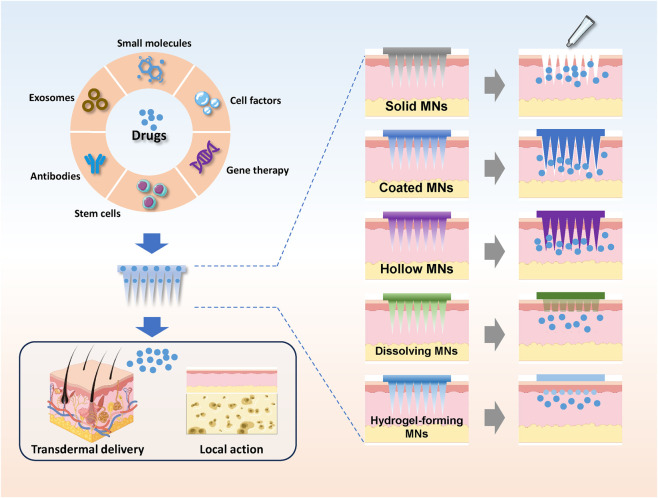
The microneedle delivery system enables the loading and delivery of various types of drug molecules.

S-MNs function primarily as pre-treatment tools. They create temporary microchannels in the skin, which are then used for drug application via methods like passive diffusion or electroporation. While their high mechanical strength is a distinct advantage, the requirement for a two-step process limits their convenience. Resembling miniature hypodermic needles, H-MNs feature a hollow interior that allows liquid formulations to be precisely injected into the dermal layer via micro-pumps or osmotic pressure. They are particularly suitable for high-dose or continuous infusion delivery. However, manufacturing complexity and the potential for clogging present technical challenges. C-MNs typically consist of an inert core (e.g., metal or rigid polymer) uniformly coated with a drug-matrix mixture. Upon skin implantation, the coating rapidly dissolves or diffuses into the subcutaneous tissue, leaving the needle body intact for subsequent removal. This design is ideal for the rapid release of small molecules, peptides, or vaccines. These are composed entirely of water-soluble or biodegradable polymers (e.g., hyaluronic acid, polyvinyl alcohol) mixed with the active drug. Upon application, the microneedles completely dissolve in the interstitial fluid, releasing their cargo. The primary advantages of D-MNs are their residue-free delivery and exceptional biosafety, making them an ideal platform for precise, dose-controlled release. HF-MNs represent a novel, “capsule-like” design. Composed of hydrophilic polymers, these needles absorb interstitial fluid upon penetration and swell *in situ* to form a hydrogel lattice. The drug, which is pre-encapsulated within the matrix, is then released in a sustained manner. By combining residue-free application with controlled-release capabilities, HF-MNs are particularly suitable for chronic disease treatments requiring prolonged therapeutic effects (Figure 3).

### 3.2 Materials selection and biocompatibility considerations

The performance, penetration efficiency, and safety of MNs depend strongly on material selection. Polymers are the most widely used class of materials. These include natural polymers, such as hyaluronic acid and chitosan, and synthetic polymers, such as polylactic acid (PLA), polyvinyl alcohol (PVA), and polyglycolic acid (PGA) (Lu et al., 2024). Biodegradable polymers like PLA are advantageous because they permit *in vivo* metabolism, eliminating the need for needle retrieval. Furthermore, their composition can be tuned to precisely control drug release kinetics (Abbasi et al., 2024).

Metals are commonly used as cores for solid or hollow MNs. Stainless steel and titanium alloys provide the high mechanical strength and sharpness necessary for efficient insertion; however, they must be removed after delivery. Ceramics and silicon offer superior hardness and can be fabricated with ultra-sharp tips, but their brittleness increases the risk of fracture, necessitating careful safety assessments. Additionally, carbon nanomaterials are increasingly used as reinforcers or components of drug-carrier composites to enhance mechanical properties or loading capacity (Razzaghi et al., 2024b).

Biocompatibility is essential for clinical translation. Ideal MN materials must support tissue repair with minimal local injury during insertion and degradation. Critically, they should not provoke significant immune reactions, such as inflammatory cell infiltration (Cao et al., 2019). Dissolving MNs are often considered the safest option because they are fully degradable and leave no sharp residues. Conversely, for non-degradable materials like metals or silicon, rigorous surface finishing and retrieval protocols are required to prevent debris retention and minimize the risk of allergic or foreign body responses.

Mechanical performance is essential for reliable and painless skin penetration by MNs. The most important parameters are insertion force, fracture resistance, and tip sharpness. Insertion force should remain within 0.05–0.3 N per needle: values below 0.05 N often result in incomplete penetration, whereas values above 0.3 N heighten pain perception. Fracture resistance requires a Young’s modulus >2 GPa for stainless-steel MNs or >0.5 GPa for polymeric MNs to prevent buckling. Tip sharpness must achieve a radius <20 µm to stay below the nociceptor activation threshold and ensure painless insertion.

Geriatric skin poses distinct challenges owing to age-related changes: elasticity declines markedly (elastic modulus falls 30%–50% in individuals >70 years), fragility increases (tear resistance decreases ∼40%), and stratum corneum thickness becomes more variable (10–40 µm versus 15–20 µm in young adults). To address these characteristics, MNs for elderly patients require specific adaptations, including shorter needle lengths (150–250 µm) that reliably breach the stratum corneum yet avoid dermal vasculature, tapered geometries that gradually reduce diameter to lower insertion force, and flexible backing materials (e.g., polyurethane films) that conform to skin curvature and movement. *Ex vivo* studies confirm the effectiveness of these modifications: dissolving MNs with 200 µm height achieved 92% successful insertion into elderly human skin, compared with 98% in younger skin, demonstrating that carefully optimized designs can largely overcome age-related barriers.

## 4 Potential of MN delivery systems for metabolic bone diseases

MN systems provide an attractive engineering platform to overcome core barriers in systemic therapy for metabolic bone diseases. These barriers include low bioavailability, gastrointestinal intolerance, and poor adherence. MN-based approaches have shown meaningful benefits in the management of rheumatoid arthritis, osteoarthritis, and osteoporosis, with a steady rise in research activity over the past 5 years. Gains in delivery efficiency, simplicity, cost-effectiveness, patient acceptance, and safety enable robust systemic exposure through a minimally invasive route. These features support the development of next-generation strategies that enrich drugs within the bone microenvironment in a targeted manner.

### 4.1 MN-enabled shifts in therapeutic paradigms for bone disorders

Because MNs cross the stratum corneum or mucosal barriers without damaging nerves or blood vessels, they are evolving from simple “transdermal conduits” into intelligent platforms that integrate precise delivery, controlled release, and local microenvironment modulation. This shift is reshaping care for bone and joint diseases. A primary example is the painless delivery of biologics. Therapy for rheumatoid arthritis (RA) often relies on monoclonal antibodies or fusion proteins targeting tumor necrosis factor alpha (TNF-α) or interleukin-6 (IL-6). While effective, subcutaneous injections can trigger needle aversion and treatment interruptions. Cao et al. demonstrated that hyaluronic acid crosslinked MNs could deliver etanercept efficiently without loss of bioactivity (Zhang X. et al., 2025). More recently, Zhang et al. integrated a dual-specific TNF-α/IL-6 receptor “fenobody” into gelatin methacrylate (GelMA) MNs. This system suppressed both nuclear factor kappa B (NF-κB) and Janus kinase/signal transducer and activator of transcription 3 (JAK/STAT3) pathways, achieving injection-like efficacy via noninvasive administration (Du G. et al., 2021).

Beyond delivery, the value of MNs extends to active immunomodulation, with demonstrated effects primarily involving targeted suppression of inflammatory pathways and cellular responses. Du et al. and Jin et al. utilized hyaluronic acid or chondroitin sulfate MNs to deliver melittin. Electrostatic binding reduced toxicity and promoted either regulatory T-cell expansion or synoviocyte apoptosis, effectively shifting the paradigm from passive dosing to active immune remodeling (Jin et al., 2025; Xia et al., 2024). Similarly, Xia et al. designed cerium/manganese oxide nanoparticles (Ce/MnO NPs) with enzyme-mimetic activity as carriers. This transdermal system delivered methotrexate (MTX) to suppress inflammation while driving macrophage polarization from M1 to M2, illustrating a compelling “drug plus carrier” dual-therapy concept (Katsumi et al., 2012). These examples highlight specific, experimentally validated immunomodulatory outcomes, such as cytokine inhibition and macrophage repolarization, though broader osteoimmune mechanisms remain under investigation and require further mechanistic delineation.

MNs are also moving beyond acting as mere “injection replacements” toward becoming long-acting, regionally targeted, and responsive designs. For osteoporosis, Katsumi et al. used tip-loaded MNs to raise the transdermal bioavailability of alendronate above 90%. The system reduced growth plate degeneration in osteoporotic rat models and avoided cutaneous irritation seen with full-length drug-loaded MNs or intradermal injection, indicating strong safety with efficient delivery (Katsumi et al., 2017; Xu et al., 2025). Xu et al. developed a core–shell MN that enabled sustained release of alfacalcidol for up to 14 days. Twice-monthly dosing matched the bone-protective effect of daily oral therapy while lowering the total systemic dose and improving trabecular structure (Mary et al., 2024). Bisphosphonates such as alendronate are often limited by gastrointestinal irritation and very low oral bioavailability (<1%). MN delivery bypasses the gastrointestinal tract, supports noninvasive absorption, and may reduce local inflammatory risk (Uddin et al., 2024). Addressing environmental and manufacturing concerns, Uddin et al. created a 3D-printed hollow MN array (μNe3dles) using a high bio-based, photo-curable resin composed of isobornyl acrylate and pentaerythritol triacrylate in a 1:1 ratio. The device showed excellent mechanical strength and penetration. In an osteoporotic mouse model, this system delivered the monoclonal antibody denosumab with faster *in vitro* release and superior *in vivo* recovery of bone mineral indices compared with subcutaneous injection (Hu et al., 2024).

A further advance is the coupling of MNs with externally triggered or self-responsive systems. Hu et al. constructed an extracorporeal shock wave (ESW)-driven nanomotor combined with dissolving MNs (ESW-NM-MN). Zoledronic acid (ZOL) was loaded into calcium phosphate nanomotors with a high Young’s modulus. After MN insertion, ESW activated nanomotor movement into deeper tissues and triggered disassembly, releasing ZOL and Ca^2+^ to suppress osteoclastogenesis and promote osteogenesis. The platform increased local bone density and reduced fracture risk *in vivo*, supporting regional precision therapy for osteoporosis (Peng et al., 2025). In another responsive design, Peng et al. engineered an ultrasound-responsive dissolving MN system (MTX-PFP-NPs@DMNs). Poly(lactic-co-glycolic acid) (PLGA) nanoparticles carrying MTX and perfluoropentane (PFP) were embedded in hyaluronic acid MNs. Ultrasound induced a PFP phase transition and cavitation that enhanced drug permeation and diffusion. The MNs dissolved within 20 min *ex vivo* and penetrated the stratum corneum efficiently. In collagen-induced arthritis (CIA) mice, ultrasound plus MNs reduced joint swelling, bone erosion, cartilage damage, and pro-inflammatory mediators, indicating a high-adherence, synergistic strategy for RA (Li et al., 2025). These examples mark a clear transition from static delivery to dynamic, stimulus-responsive therapy.

Finally, MNs can act as local microenvironment modulators, extending from skin to mucosa and specialized bone–soft tissue interfaces. Li et al. developed hydrogel MNs that penetrate gingival mucosa. In a diabetic periodontitis model, the system combined antibacterial and antioxidant actions with inhibition of JAK-STAT and NF-κB signaling, which promoted alveolar bone regeneration (Shan et al., 2025). Shan et al. achieved precise mucosal delivery of RANKL to periodontal tissue. Localized RANKL drove osteoclast formation and accelerated orthodontic tooth movement (Nguyen et al., 2025). These studies introduce innovative transmucosal strategies for early intervention in metabolism-related bone conditions. In summary, the trajectory of MN technology in MBD care is moving from better adherence to superior efficacy, and ultimately to microenvironment remodeling, based on the demonstrated outcomes above. Continued convergence of materials science, nanotechnology, and disease biology may integrate diagnostics, feedback sensing, and adaptive release within MN platforms. Such systems could enable a true “theranostic” paradigm for precise and minimally invasive orthopedics. Hypothetically, the trajectory toward a “theranostic” MN platform for bone health could involve three progressive stages: (i) Current MNs primarily serve as delivery vehicles with passive release kinetics; (ii) Next-generation systems could integrate wearable biosensors (e.g., flexible electrochemical sensors for serum calcium or bone turnover markers like CTX/P1NP in interstitial fluid) with MN patches to enable biomarker-triggered release; (iii) Future “closed-loop” platforms might combine real-time monitoring of bone microenvironment cues (e.g., local pH changes during active resorption, RANKL concentration gradients) with on-demand drug release.

### 4.2 Molecular and pharmacokinetic considerations for MN-based therapy in bone disease

Animal studies and proof-of-concept work show that MN systems can deliver many key agents for MBDs with efficient absorption, particularly for gastrointestinally labile biologics and small molecules that benefit from sustained release. Calcitonin, a 32-amino-acid peptide hormone, is rapidly degraded by digestive enzymes after oral dosing and thus has poor bioavailability, while injections suffer from low adherence. Dissolving MN arrays fabricated from biodegradable polymers such as PLGA or HA have been used to encapsulate calcitonin. These MNs preserve peptide integrity, enable efficient transdermal uptake, and produce pharmacokinetic profiles in rodents comparable to subcutaneous injection. They successfully raise systemic exposure and suppress osteoclast activity, supporting feasibility for systemic antiresorptive therapy (Sim et al., 2022).

Significant progress has also been made with parathyroid hormone analogs. MNs have achieved once-weekly administration of teriparatide acetate (TA). Through formulation optimization (incorporating hyaluronic acid and trehalose) and a bilayer structure design (bottom layer composed of pure hyaluronic acid, top layer loaded with TA), a TA-DMN patch demonstrated excellent puncture capability and rapid drug release (87.6% released within 5 min) in *ex vivo* porcine skin. In rats, this system achieved 66.9% relative bioavailability. Plasma drug concentrations showed a positive correlation with patch quantity, indicating that this system maintains TA activity while demonstrating potential as a patient-friendly delivery platform for long-acting osteoporosis therapy (Zhang et al., 2020). Similarly, two dissolving MN arrays (sCT-DMNA-1 and sCT-DMNA-2) for salmon calcitonin (sCT) further illustrated this concept. Trehalose improved sCT stability under heat and humidity. *In vivo*, the patches reached ∼70% relative bioavailability and enhanced transdermal delivery while preserving mechanical performance, offering a safe, efficient, and low-pain alternative (Oh et al., 2022). Additionally, teriparatide MNs formulated with sucrose and carboxymethyl cellulose (CMC) increased the maximum plasma concentration (Cmax) and area under the curve (AUC) in rats. *In vitro* skin-diffusion data predicted *in vivo* pharmacokinetics, underscoring that MN dissolution rate is a key lever to tune TA transdermal pharmacokinetics (Yang et al., 2024).

Osteoporosis is a prevalent MBD marked by microarchitectural deterioration and fracture risk. Minodronic acid (MA) is a potent antiresorptive agent, but oral dosing has very low bioavailability, notable adverse effects, and poor adherence. MA-loaded dissolving MNs (MA-MNs) improved pharmacokinetics after load optimization. At a minimal dose of 224.9 μg, MA-MNs increased peak concentration and bioavailability by 9-fold and 25.8-fold, respectively, versus oral MA. Furthermore, they prolonged half-life with more stable plasma levels (Salameh et al., 2024). For nutrition-deficiency related bone disease, long-term, steady supplementation is essential. MN systems address solubility-driven barriers to transdermal vitamin delivery (Hamed et al., 2024). Embedding vitamins within the matrices of coated or dissolving MNs made from water-soluble polymers such as PVA and PVP avoids inter-individual variability in gastrointestinal absorption and batch-to-batch issues seen with oral products. Polymer composition can be tuned to modulate release, enabling sustained delivery over days to weeks (Kim et al., 2025; Pramoda et al., 2025).

MN-mediated systemic exposure is highly dependent on molecular properties and formulation design. Small hydrophilic molecules (<500 Da) primarily enter systemic circulation via dermal capillaries, while larger molecules (>5 kDa) and particulate carriers show preferential uptake by dermal lymphatic vessels (Farooq et al., 2025). This distinction critically impacts pharmacokinetics: lymphatic uptake delays systemic appearance (Tmax 2–6 h vs. 0.5–2 h for capillary route) but may enhance bioavailability for molecules susceptible to first-pass metabolism. For bone-targeted biologics delivered via MNs, dermal metabolism presents an underappreciated barrier. Enzymes such as matrix metalloproteinases (MMP-2/9) and cathepsins in the dermal extracellular matrix can degrade peptide therapeutics before systemic entry. Strategies to mitigate this include (i) incorporation of protease inhibitors (e.g., aprotinin): in MN matrices, (ii) PEGylation of therapeutic peptides to sterically hinder enzymatic access, and (iii) rapid-dissolving formulations that minimize dermal residence time. Relative bioavailability versus oral dosing must be distinguished from absolute bioavailability: while MN delivery of alendronate achieved >90% relative bioavailability versus IV injection in rats (Katsumi et al., 2017), absolute bioavailability versus subcutaneous injection remains formulation-dependent (typically 60%–85% for dissolving MNs versus 100% for SC injection).

### 4.3 Combining MNs with bone-targeted carriers for precise enrichment and synergy

MN technology can breach skin or mucosal barriers and enable noninvasive delivery of macromolecules. After entry, however, drugs can still undergo nonspecific distribution with insufficient accumulation in bone and joint tissues, which often have relatively limited perfusion. To address this gap, MN platforms are being integrated with active targeting carriers to create an “entry via MNs, navigation via targeting” paradigm. This approach increases local drug concentration at lesions and enables multi-component, multi-pathway synergy.

Decorating drugs or carriers with affinity ligands enhances binding to bone or joint tissues (Liu T. et al., 2024). Several affinity motifs have been incorporated into MN systems. Liu et al. built MTX@PMs MNs using hyaluronic acid (HA) as a CD44 ligand to guide MTX–loaded polymeric micelles toward activated macrophages in inflamed joints of RA. This strategy improved efficacy and reduced systemic toxicity (Cao et al., 2021). Similarly, Cao et al. conjugated a high-affinity DEK-targeting aptamer (DTA6) to cholesterol and embedded it in hydrogel MNs. The platform systemically targeted multiple inflamed joints inhibited neutrophil extracellular trap (NET) formation, and protected joint structure in CIA models, overcoming the limited reach of intra-articular injection for polyarticular disease (Lei et al., 2024). The Arg-Gly-Asp peptide, which binds integrin receptors enriched in bone-remodeling microenvironments, is another promising bone-targeting ligand with potential use in osteoporosis or fracture repair MNs (Lin et al., 2023).

Beyond ligand functionalization, the introduction of biomimetic and smart responsive carriers further enhances the targeting capabilities and therapeutic dimensions of microneedle systems. Lin et al. coated nonsteroidal anti-inflammatory drug nanoparticles with neutrophil membranes and loaded them into MNs. The biomimetic cloak provided inflammation homing and cytokine adsorption, enabling RA lesion “homing” with local cyclooxygenase-2 inhibition and concurrent microenvironment modulation (Li et al., 2024). Li et al. loaded polydopamine-modified exosomes (PDA@Exo) into MNs. By activating the PI3K/Akt/mTOR pathway, the system cleared reactive oxygen species, sustained cartilage homeostasis, promoted M2 macrophage polarization, and supported bone regeneration, yielding disease-modifying effects in osteoarthritis models (Zhou et al., 2025). Zhou et al. used D-RADA16 self-assembling peptides to form an extracellular matrix (ECM)-like nanonetwork *in situ* within joints, creating a “cellular safe house” that blocked mitochondrial DNA leakage and the cGAS–STING pathway (An et al., 2022). This strategy reduced inflammation and cartilage degeneration *in vitro* and *in vivo*, suggesting a precise, durable, and patient-friendly noninvasive option for chronic inflammatory joint disease.

While bone-targeting ligands, such as bisphosphonates, aspartic acid oligomers, and RGD peptides, enhance skeletal accumulation after systemic administration, their application following MN delivery necessitates pharmacokinetic reevaluation. MN delivery generally produces lower peak plasma concentrations but extends exposure time (with increased AUC0-24 h) relative to bolus injection, a profile that may in fact benefit bone targeting since hydroxyapatite-binding ligands rely on sustained exposure for accumulation at remodeling sites rather than high Cmax. As mentioned above, computational modeling showed that for alendronate-conjugated nanoparticles, MN delivery, with 50% lower Cmax but twice the half-life, yielded bone uptake comparable to intravenous injection, owing to prolonged circulation. Nevertheless, significant knowledge gaps persist, including the predominance of intravenous administration in most bone-targeting studies coupled with a scarcity of MN-specific pharmacokinetic data, potential alterations to ligand conformation or binding affinity due to dermal first-pass effects, and reduced targeting efficiency from competition with endogenous calcium-binding proteins in interstitial fluid.

Notably, multi-drug synergism and differentiated release have become key pathways for enhancing therapeutic efficacy. An et al. created a detachable MN with spatially separated materials, enabling sustained release of tocilizumab (TCZ) and rapid release of a TNF aptamer. Dual blockade of IL-6R and TNF outperformed monotherapy in CIA models (Zheng et al., 2024). Zheng et al. integrated dissolving MNs loaded with melittin and a diclofenac sodium transdermal patch to combine anti-inflammatory and immunoregulatory actions with analgesia. The system alleviated paw swelling and reduced synovial, joint, and cartilage injury in RA models, highlighting potential as a combination modality (Liu T. et al., 2025).

**FIGURE 4:**
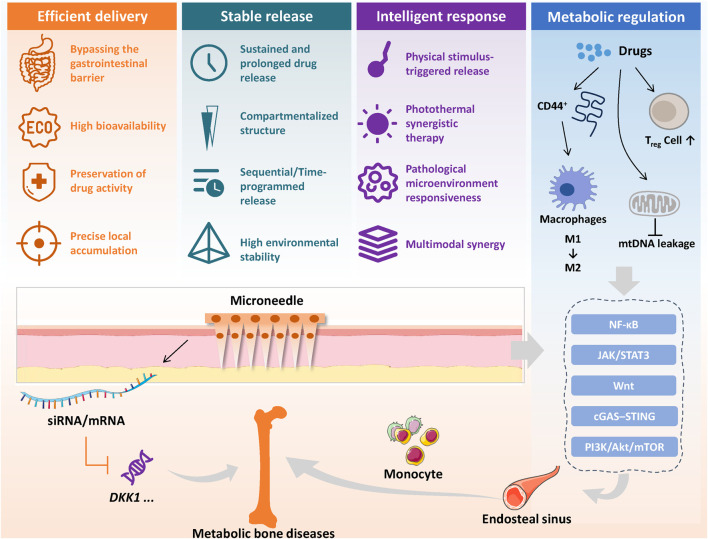
Advantages of microneedle delivery systems in treating metabolic bone diseases.

Furthermore, the introduction of active delivery mechanisms is breaking through the limitations of traditional microneedles that rely on passive diffusion. Liu et al. embedded a sodium bicarbonate/citric acid gas-generating system inside MNs. Carbon dioxide bubbles generated impulsive thrust that drove deeper drug penetration and improved transdermal efficiency (Meng et al., 2024). Meng et al. designed a near-infrared responsive PDA/GelMA MN that penetrates the annulus fibrosus and delivers combined photothermal and pharmacologic therapy. The platform mounted an “offensive” anti-inflammatory action extracellularly and induced intracellular heat-shock responses for “defense,” restoring ECM synthesis and biomechanics in intervertebral disc degeneration models (Maguire, 2022). In summary, combining MNs with bone and joint-targeted carriers has shifted the field from a single-mode delivery tool to intelligent platforms that integrate spatial targeting, temporal control, pathway synergy, and microenvironment remodeling (Figure 4). As ligand libraries expand, responsive materials improve, and mechanisms of multi-component cooperation are clarified, such “entry-and-navigation” systems are poised to deliver precise, durable, and individualized therapy for bone and joint diseases.

## 5 Challenges and future directions

MN systems demonstrate significant potential to improve adherence and systemic exposure for metabolic bone disease therapies. However, translating these platforms from proof-of-concept studies to large-scale clinical use still faces substantial technical, manufacturing, and clinical hurdles.

### 5.1 Constraints of micro-scale geometry and drug kinetics

The primary technical challenge stems from the functional limits imposed by the small geometry of microneedles. The effective volume of an MN is typically in the nanoliter to picoliter range. Yet, systemic indications often require large doses or the delivery of high–molecular weight biologics, such as antibodies. Achieving high drug concentrations within such limited space is difficult. The dose ceiling represents a fundamental constraint for MN translation to MBDs. Typical MN arrays (1 cm^2^) contain 100–400 needles with individual volumes of 10–100 nL, yielding total payload capacity of 1–40 mg depending on drug solubility and matrix composition. This poses challenges for high-dose MBD therapies: denosumab (60 mg/month), zoledronic acid (5 mg/year IV but requires higher doses for transdermal delivery due to incomplete absorption), and teriparatide (20 µg/day but requires chronic administration). Three engineering strategies show promise: (i) Tip-loaded MNs concentrate drug in the distal 20%–30% of needle length that penetrates skin, increasing effective payload by 3–5× while maintaining mechanical integrity; (ii) Multi-layer MN architectures with drug-loaded core and structural shell enable 2–3× higher loading without compromising insertion force; (iii) Larger patch formats (4–9 cm^2^) with optimized skin adhesion can deliver clinically relevant doses but face patient acceptability challenges, particularly for geriatric populations with fragile skin. Computational modeling suggests that for denosumab delivery, a 4 cm^2^ MN patch with 5% (w/w) drug loading in a rapidly dissolving matrix could achieve therapeutic plasma concentrations, though this requires validation in large animal models with human-relevant skin thickness. Furthermore, high loading can compromise the structural stability of peptides and proteins, leading to aggregation or denaturation and a subsequent loss of bioactivity. Future work must therefore focus on developing nanostructured carriers with ultra-high loading efficiency and built-in molecular stabilization mechanisms.

A second critical issue is controlling release kinetics. Many MBD therapies, particularly osteoanabolic agents, require sustained or pulsatile release over weeks to months. Conversely, dissolving MNs often exhibit rapid burst release, which exhausts the therapeutic payload in a short time. Achieving near zero-order kinetics will likely require advanced polymer engineering. Potential solutions include the development of multilayer or porous MN architectures, the integration of microfluidic channels, or encapsulation within microcapsules or nanocapsules with staggered degradation rates to precisely tune diffusion and dissolution.

In addition to drug loading and release, mechanical reliability is paramount. MNs must be strong enough to pierce the stratum corneum reliably, yet not so rigid that insertion becomes painful. Moreover, inter-individual differences in skin thickness, elasticity, and hydration produce significant variability in penetration depth and absorption rates. At an industrial scale, product reliability depends on the tight control of tip sharpness, needle height uniformity, and dose content uniformity across batches. Ensuring these parameters are maintained during mass manufacturing is essential for clinical success.

### 5.2 Clinical translation bottlenecks: safety, adherence, and regulatory standards

Clinical translation requires a systematic evaluation of patient safety, long-term efficacy, and manufacturing practices. Although MNs are minimally invasive, repeated or prolonged use can increase the risk of local irritation, erythema, or inflammatory reactions. This is especially relevant when non-degradable materials are used. For dissolving or hydrogel MNs, the rate of skin-barrier recovery after complete needle degradation serves as a key safety endpoint. Therefore, robust sterilization and biocompatibility testing are essential to minimize infection risks.

Particular attention must be paid to the biological risks associated with improper application. For instance, frequent microneedling combined with topical bone-marrow–derived cytokines for cosmetic skin care carries potential hazards. Such procedures are often promoted by non-dermatology clinicians or non-physician providers who may lack formal training in cutaneous immunology. MNs can trigger sterile inflammation. If asepsis is inadequate, the dense skin microbiota may enter the dermis through microchannels and amplify local or systemic inflammation. Furthermore, pro-inflammatory bone-marrow–derived cytokines can sustain chronic inflammation. This contrasts with adipose- or skin-derived mesenchymal stromal cell products, which may aid resolution. Consequently, improper use can disrupt cutaneous homeostasis (Meng et al., 2024).

Beyond biological safety, practical usability determines real-world adoption. For MN patches designed to remain in place for hours to days, skin adhesion must withstand routine activities such as movement, perspiration, and bathing to ensure continuous dosing. Patient acceptance also depends on perceived minimal invasiveness, comfort, and ease of self-administration. Ultimately, MN platforms must demonstrate a clear quality-of-life advantage over oral or injectable therapy to achieve widespread adoption.

Finally, manufacturing presents distinct complexities. Current MN fabrication methods, including micromolding and photolithography, lack harmonized guidance under Good Manufacturing Practice (GMP). Regulatory expectations for this novel delivery class are still evolving. This includes undefined standards for quality attributes, *in vitro* and *in vivo* performance assays, and requirements for long-term stability. These gaps create significant uncertainty for industrial scale-up.

### 5.3 Clinical translation status and regulatory pathways

To date, no MN-based therapies have received regulatory approval specifically for metabolic bone diseases. However, the clinical pipeline provides valuable context: (i) Vaxxas’ high-density MN patch for influenza vaccine completed Phase I/II trials (NCT03251874) demonstrating safety and immunogenicity; (ii) Zosano Pharma’s Qtrypta® (zolmitriptan MN patch) received FDA approval in 2020 for migraine, validating MN technology for systemic delivery; (iii) Micron Biomedical’s dissolving MN vaccine platform entered Phase I trials in 2023 (NCT05687391). For bone-related applications, preclinical studies dominate the landscape. As mentioned above, MN loaded with targeted drugs has shown superior efficacy in rodent osteoporosis models compared to oral/injection routes, but has not yet progressed to IND-enabling studies.

Key translational barriers specific to MBDs include: (i) dose requirements (e.g., denosumab requires 60 mg monthly; current MNs typically deliver <10 mg/patch), (ii) need for chronic administration (>5 years for osteoporosis), and (iii) absence of established regulatory guidelines for MN-biologic combination products targeting skeletal endpoints. The FDA’s 2023 draft guidance on “Transdermal and Topical Delivery Systems” provides partial framework, but bone-specific endpoints (BMD changes, fracture risk reduction) require novel clinical trial designs.

## 6 Conclusion

MBDs stem from complex systemic metabolic dysregulation. Historically, clinical effectiveness has been constrained by two primary obstacles: the poor oral bioavailability of biologics and low patient adherence to long-term injection regimens. This review highlights how MN systems offer a transformative solution. They provide a minimally invasive, patient-friendly transdermal route capable of delivering peptides, antibodies, and nucleic acids efficiently. By bypassing first-pass hepatic metabolism and gastrointestinal degradation, MNs significantly increase effective systemic exposure. Ultimately, with their low invasiveness, high adherence, and favorable pharmacokinetics, MN platforms overcome the limitations of conventional dosing and demonstrate strong potential for clinical translation.

The physiological disconnect between dermal triggers and bone microenvironment cues constitutes a fundamental limitation of current stimuli-responsive microneedle (MN) designs. For instance, ultrasound-responsive MNs, depend on external energy input rather than endogenous disease biomarkers; although clinically feasible, this approach demands ongoing patient compliance with an external device. Similarly, pH-responsive systems are engineered to detect acidic microenvironments (typically pH 5.5–6.5) prevalent in inflamed joints, yet they are less effective in systemic bone resorption sites where pH alterations remain subtle (around 7.2–7.4). Enzyme-responsive MNs targeting MMP-9 activation also carry the risk of premature triggering in dermal wounds or inflamed skin before the system can reach deeper bone targets. A truly bone microenvironment-responsive MN platform would need to sense specific local cues, such as RANKL concentration gradients (exceeding 100 pg/mL at active resorption sites compared to below 20 pg/mL in quiescent bone), dynamic calcium fluxes associated with osteoclast activity, or changes in sclerostin levels that reflect osteocyte function. At present, however, none of these key bone-specific biomarkers are detectable by sensors integrated into dermal MNs.

Looking ahead, the development of MNs will move beyond simple transdermal transport toward integrated delivery loops that are intelligent, precise, and trackable. A key priority is the deep integration of bone-targeted nanocarriers with MNs. This approach aims to enrich therapeutic agents specifically within the pathological bone-remodeling microenvironment. Furthermore, future systems must incorporate multi-level, bioresponsive mechanisms to enable on-demand release guided by *in vivo* signals. Finally, coupling MNs with wearable sensors to monitor bone-turnover biomarkers in real time will support truly personalized, feedback-controlled dosing. Through the continued cross-disciplinary convergence of engineering and biology, MNs are well-positioned to transform the treatment paradigm for MBDs, improving long-term outcomes and quality of life for millions of patients.
